# Robotic natural orifice specimen extraction surgery versus traditional robotic-assisted surgery (NOTR) for patients with colorectal cancer: a study protocol for a randomized controlled trial

**DOI:** 10.1186/s13063-021-05077-z

**Published:** 2021-02-06

**Authors:** Rui Luo, Fangfang Zheng, Haobo Zhang, Weiquan Zhu, Penghui He, Dongning Liu

**Affiliations:** 1 The First Clinical Medical College of Nanchang University, Nanchang, 330000 China; 2 Department of Cardiology, The Third Hospital of Nanchang, Nanchang, 330000 China; 3 Department of General Surgery, The First Affiliated Hospital of Nanchang University, Nanchang, 330000 China

**Keywords:** Natural orifice extraction surgery, Randomized controlled trial, Robotic surgery, Colorectal cancer

## Abstract

**Background:**

Natural orifice specimen extraction surgery for colorectal cancer has been introduced in order to reduce the abdominal incision, demonstrating major development potential in minimally invasive surgery. We are conducting this randomized controlled trial to assess whether robotic NOSES is non-inferior to traditional robotic-assisted surgery for patients with colorectal cancer in terms of primary and secondary outcomes.

**Method/design:**

Accordingly, a prospective, open-label, randomized controlled, parallel-group, multicenter, and non-inferiority trial will be conducted to discuss the safety and efficacy of robotic natural orifice extraction surgery compared to traditional robotic-assisted surgery. Here, 550 estimated participants will be enrolled to have 80% power to detect differences with a one-sided significance level of 0.025 in consideration of the non-inferiority margin of 10%. The primary outcome is the incidence of surgical complications, which will be classified using the Clavien-Dindo system.

**Discussion:**

This trial is expected to reveal whether robotic NOSES is non-inferior to traditional robotic-assisted surgery, which is of great significance in regard to the development of robotic NOSES for patients with colorectal cancer in the minimally invasive era. Furthermore, robotic NOSES is expected to exhibit superiority to traditional robotic-assisted surgery in terms of both primary and secondary outcomes.

**Trial registration:**

ClinicalTrials.govNCT04230772. Registered on January 15, 2020.

## Introduction

Laparoscopic natural orifice specimen extraction surgery (NOSES) is currently a research hotspot. Compared to traditional laparoscopic radical colorectal cancer resection, laparoscopic NOSES possesses advantages in reducing postoperative pain, incidence of infection at the incision site, and other related complications [1–3]. Moreover, it improves surgical esthetics and reduces gastrointestinal recovery time so patients may receive enteral nutrition earlier [4, 5]. However, various problems and technical bottlenecks in the development of NOSES of colorectal cancer exist [6, 7] including (1) the operation time of NOSES is longer; (2) the technical requirements of NOSES for the surgical team are relatively higher; (3) when extracting the specimen through the anus or vagina, squeezing the tumor, which may lead to tumor cell dissemination, is unavoidable; (4) to reconstruct the digestive tract, an opening on intestine is required but may easily cause pelvic and abdominal infections; and (5) a potential risk of vagina and anus strain injury is present when specimens are extracted through these structures.

The aforementioned ideas are built according to the current development of laparoscopic NOSES in colorectal cancer. No studies pertaining to robotic NOSES have been previously discussed. However, innumerable merits in robotic surgery have been reported including (1) filter physiological trembling; (2) magnification and provision of a three-dimensional view of the surgical field of view; and (3) accurately cutting, separating, and suturing even in narrow pelvic spaces [8–10]. These technical advantages have created satisfactory conditions in shortening the operation time and reducing the postoperative complication rate such as anastomotic fistula, abdominal infection, and abdominal hemorrhage [11]. To our knowledge, no trials in regard to robotic NOSES for colorectal cancer have been conducted. Hence, this randomized controlled trial is being performed to assess whether robotic NOSES is non-inferior to traditional robotic-assisted surgery for patients with colorectal cancer in terms of primary and secondary outcomes.

## Methods/design

### Objectives

By comparing traditional robotic-assisted surgery, we aim to evaluate the perioperative safety and efficacy of robotic natural orifice specimen extraction surgery.

### Trial design

This study is a prospective, open-label, randomized controlled, parallel-group, multicenter, and non-inferiority trial conducted in seven academic hospitals in China. The hospitals involved are shown in Table 1.

**Table 1:** Collaborator hospitals, locations, principle investigator/collaborator and anticipated participants

Hospitals	Locations	Names	Role in this study	Anticipated participants
The First Affiliated Hospital of Nanchang University	China, Jiangxi	Taiyuan Li,	Principle investigator	130
		Dongning Liu	Steering committee	
		Division of data management	Data management team	
The Second Xiangya Hospital of Central South University	China, Hunan	Hongliang Yao	Collaborator	70
Nanfang Hospital, Southern Medical University	China, Guangdong	Yanan Wang	Collaborator	70
First Affiliated Hospital of Gannan Medical College	China, Jiangxi	Xiangfu Zeng	Collaborator	70
Zhongshan Hospital, Fudan University	China, Shanghai	Ye Wei	Collaborator	70
Chinese PLA General Hospital (301 Hospital) China	China, Beijing	Baoqing Jia	Collaborator	70
The Second Affiliated Hospital of Nanchang University	China, Jiangxi	Shengxun Mao	Collaborator	70

### Inclusion criteria

The inclusion criteria are as follows:

1. Age ≥ 18 and ≤ 80 years;
2. Performance status of 0 or 1 on ECOG (Eastern Cooperative Oncology Group) scale;
3. Histological or cytological confirmation of colorectal adenocarcinoma;
4. High rectal and sigmoid cancer with the lower margin of the tumor greater than 10 cm from the anal dentate line;
5. T1-3N0M0 at preoperative evaluation according to the American Joint Committee on Cancer (AJCC) Cancer Staging Manual 8th Edition, transvaginal NOSES procedure with a specimen with a circumferential diameter of < 5 cm;
6. Preoperative examination did not suggest distant metastasis, implantation, or invasion of adjacent organs;
7. Cardiopulmonary liver and kidney function can tolerate surgery; and
8. Written informed consent for participation in the trial.

### Exclusion criteria

The exclusion criteria are as follows:

1. Not suitable for robot laparoscopic surgery;
2. The tumor is too large to be pulled out through the anus or vagina;
3. Simultaneous multiple primary cancers;
4. Emergency surgery due to complication (bleeding, obstruction, or perforation) caused by primary cancer; and
5. Women with acute gynecological infections, vaginal deformities, unmarried and infertile women, and women who are married and plan to get pregnant.

### Withdrawal criteria

The withdrawal criteria are as follows:

1. Postoperative pathology confirmed the tumor is pT4 or N1–2;
2. Intraoperative exploration or postoperative pathology confirmed distant metastasis;
3. During the treatment period, the patients received other therapies other than this study regimen;
4. Intraoperative exploration requires combined visceral resection;
5. Postoperative pathology confirmed non-rectal adenocarcinoma; and
6. After being enrolled in the study, the patients were required to be withdrawn from the study cohort for various reasons.

### Recruitment and participants

**Fig. 1:**
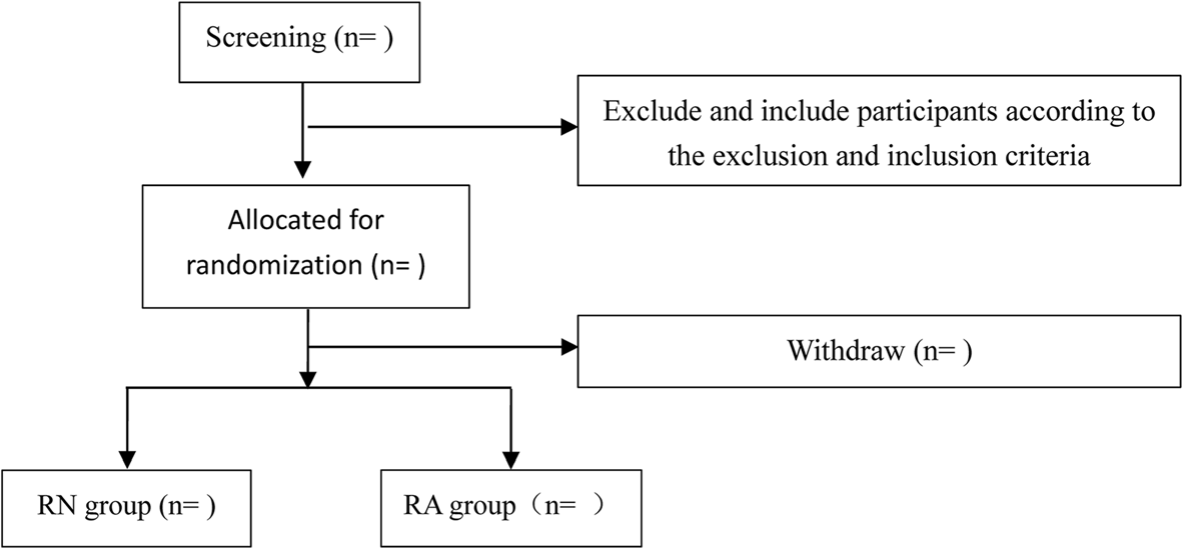
Consolidated Standards of Reporting Trials (CONSORT) flow diagram

Eligible patients will be recruited from the outpatient department or clinical wards consecutively with written consent in the listed hospitals. Their medical history will be kept in the medical record system. Before signing the surgery consent form, all participants will have the objectives, benefits, and risks of the operation as well as their rights and responsibilities explained to them. Patients who are assessed as eligible will be randomly allocated to the RN (robotic natural orifice specimen extraction surgery) and RA (traditional robotic-assisted surgery) group. The allocated participants in each group will undergo corresponding modus operandi detailed in the “Intervention” section. All participants or their legal representatives will sign the consent for operation. On the consent form, participants will be asked if they agree to have their data used should they choose to withdraw from the trial. Participants will also be asked for permission in order to share relevant data with collaborators taking part in the research or from regulatory authorities, where relevant. The enrollment and randomization flow chart is shown in Fig. 1.

### Sample size

A previously published study observed that the complication rate of robotic-assisted colorectal surgery is 17% [12]. According to the retrospective analysis in the medical record system at our institution, the complication rate of traditional robotic-assisted surgery is around 15%. As for the non-inferiority margin, no published studies can be referenced to our knowledge. After a close discussion with the investigators involved in this trial, the margin was set to 10%. We assume that this trial has 80% power (1-β) to detect the complication rate difference between the experimental and control group and a one-sided significance level of 0.025(α = 0.025). Accordingly, 402 participants in total are needed (201 per group). Considering that the overall dropout rate is 20%, we calculated that 503 participants should be enrolled in the study. Finally, 550 participants were recruited in this trial. The sample size was calculated using PASS 11 (version 11.0.7).

### Randomization and blinding

Stratified block randomization was performed by the Division of Data Management with a block size of 4. The assignment list was generated by a computer program in a block of four and was stratified by cT stage and hospital, which will be sent to the corresponding collaborators of each hospital via e-mail. Participants will then be randomly assigned to an experimental and control group with a 1:1 ratio according to the assignment list. The Division of Data Management will be blinded to the medical data except cT stage and hospital to avoid nonrandom allocation. The patients and the surgeons will not be blind to the allocation.

### Intervention

**Fig. 2:**
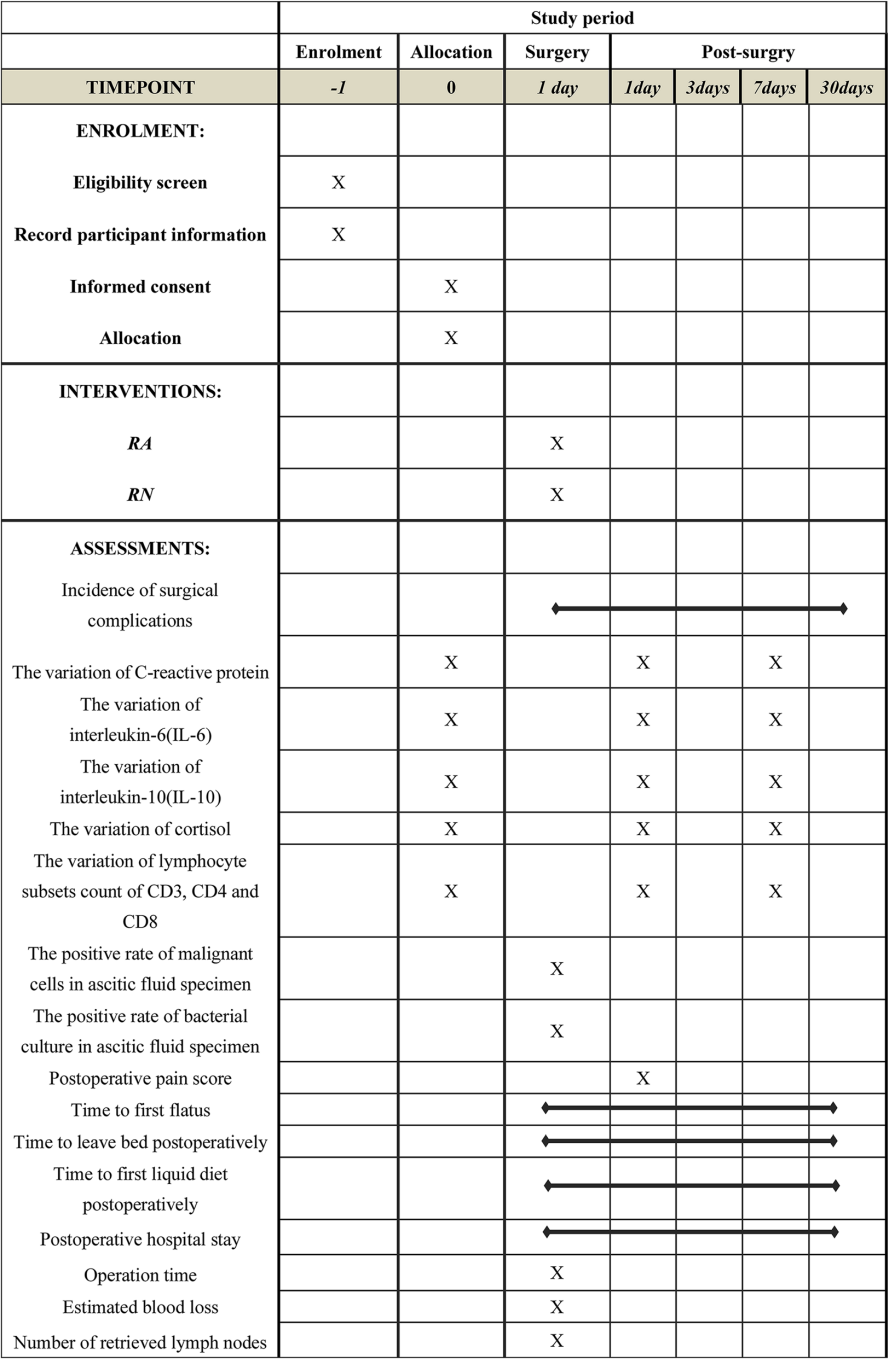
Schedule of assessment. CD3 cluster of differentiation 3, CD4 cluster of differentiation 4, CD8 cluster of differentiation 8

Patients will be randomly allocated into the RA (traditional robotic-assisted surgery) group and RN (Robotic natural orifice specimen extraction surgery) group. The specimen will be extracted through the rectum and vagina [13, 14]. In terms of female participants, in principle, extracting the specimen through rectum will be prioritized. This is a surgical trial; there are no strategies for patients to improve adherence. All the surgeons included in this study perform more than 100 robotic gastrointestinal surgeries a year. In order to make surgeons adhere to intervention protocol, critical surgical nodes will be photographed. And these photos will be submitted to the Division of Data Management. The Research Committee will check the surgical quality. The non-adherent cases will be excluded from per-protocol analysis. The schedule of this trial is shown in Fig. 2.

### Traditional robotic-assisted surgery

Patients will be performed traditional robotic-assisted colorectal resection following the total mesorectal excision(TME) principle. All patients are intubated under general anesthesia in the lithotomy position. We employ the 5-hole trocar layout. The navel is used to explore the abdominal cavity, and two operation holes are established on the right and left lower abdomen. The peritoneum is incised at the junction of the sigmoid mesocolon and the pelvis to find the inferior mesenteric vessels. Clamp and cut off the inferior mesenteric vessels while the left colonic vessels are kept as far as possible, with the surrounding lymph nodes being thoroughly cleaned. After entering the Toldts gap, the naked sigmoid colon and rectum are freely exposed outward and downward, and the rectum is cut off with a linear cutter stapler and cartridge approximately 3 to 5 cm under the lower edge of the tumor. The abdomen is then entered layer-by-layer and the incision protector is placed through a midline abdominal incision with a length of 5 to 6 cm. Afterward, the proximal intestine is removed, and a specimen is cut off from the colonic mesocolon 10 to 15 cm upper the tumor. After disinfecting the cut edge, the intestine is placed into the stapler holder and undergoes an external purse-string suture into the abdominal cavity. Pneumoperitoneum is then reestablished, and the stump of the intestine is flushed from the anus. Then, the stapler is placed, and the end-to-end anastomosis is completed under the endoscope. Finally, a rubber drainage tube is placed and fixed on the left abdominal wall next to the anastomosis from the trocar hole.

### Robotic natural orifice specimen extraction surgery via rectum

Patients will receive robotic colorectal resection with natural orifice specimen extraction surgeries through the rectum or vagina. After general anesthesia, the 5-hole method is used in the lithotomy position with the right thigh placed horizontally. After cutting the first incision in the mesorectum at about 3–5 cm below the sacral headland, the Toldts gap is then entered, freeing the root of the inferior mesenteric vessels and separating the bare inferior mesenteric vessels layer-by-layer with a pre-cut line, which is then ligated and cut. The mesorectum is freed, after which the avascular area of the sigmoid mesocolon is opened, while the rectal mesentery is freed downward and outward to the bifurcation of the left common iliac and separated downward along the anterior iliac gap. After the inferior hypogastric plexus is exposed, an ultrasonic knife is used to carry out separation at a uniform speed along the nerve surface. This process simultaneously frees the left and right sides of the rectum 3–5 cm below the tumor. After determining the location of the tumor, intestinal wall nakedness is performed within a range of about 3 cm within 5 cm below the tumor. The sigmoid colon is then exposed for about 2 cm, after which the sigmoid mesenteric vessels are ligated and cut and the pre-cut line is determined. The assistant then fully expands and washes the anus, and an iodine gauze ball is placed under the tumor to indicate the intestinal incision. After the intestinal incision is made, the assistant places the stapler holder from the anus into the abdominal cavity through the intestinal incision for backup. At the same time, a longitudinal incision is made on the intestinal wall above the tumor. The iodine gauze is sterilized and inserted into the intestine through the longitudinal incision. The intestinal canal is then cut and closed with a linear cutter stapler and cartridge above the longitudinal incision. The rectum is subsequently severed using an ultrasonic knife at the incision below the tumor. The assistant then places the specimen into the protector through the anus by the oval forceps outside and clamps the specimen out of the anus using the oval forceps. The cutter stapler and cartridge close the stump of the distal rectum canal, and the annular stapler is then inserted through the anus. The stapler holder is then docked with the body to complete the end-to-end anastomosis. Finally, a water injection test is performed to determine the presence of bleeding or leakage, after which a rubber drainage tube is placed in the pelvic cavity.

### Robotic natural orifice specimen extraction surgery via vagina

Following general anesthesia, the five-hole method is adopted in the lithotomy position with the right thigh placed horizontally. After cutting the first incision in the mesorectum 3–5 cm below the sacral headland, the Toldts gap is entered, after which the root of the inferior mesenteric vessels is freed. Being careful to protect the ureter while separating the bare inferior mesenteric vessels layer-by-layer with a pre-cut line, they are then ligated and cut, after which the mesorectum is freed. Afterward, the avascular area of the sigmoid mesocolon is opened. The assistant lifts the back of the mesorectum and frees it downward and outward 3–5 cm below the tumor. Then, the right rectal separation and the sigmoid colon and left rectal separation are performed. The mesorectum is transrectally cut about 5 cm below the tumor, baring about 2 cm of the intestine canal. The assistant instructs the surgeon to take a 3–4 cm incision in the posterior vaginal fornix by a bladder hook trans-vaginally. The stapler holder is then delivered into the abdominal cavity through the incision. The intestine canal is cut longitudinally 1 cm below the sigmoid rectal pre-cut line above the tumor, and the stapler holder is placed into the intestine through the incision, after which the sigmoid colon and naked area under the tumor are transected with a linear cutter stapler and cartridge. The assistant uses an oval forceps to deliver a sterile plastic protector into the abdominal cavity through the vagina. Together with the surgeon, the specimen is placed in the protector and then pulled out of the body using the oval forceps. An annular stapler is then placed through the anus, and the stapler holder is docked with the body in order to complete the end-to-end anastomosis. Finally, the water injection test was done to check for the presence of bleeding or leakage. After venting abdominal cavity gas, the incision was sutured in the posterior vaginal fornix with a barbed suture, and a rubber drainage tube was placed in the pelvic cavity.

Operation video, key part photos, surgical resection specimens, and other imaging materials should be kept during the operation.

### Postoperative management

Patients in both groups will receive the same care after surgery, and adjuvant therapy will be implemented in accordance to the NCCN guidelines for colon and rectal cancer.

### Primary outcomes

Incidence of surgical complications 30 days postoperative. The classification of surgical complications will be classified using the Clavien-Dindo system.

### Secondary outcomes

The following secondary outcomes will be measured: (1) Surgical stress response (i.e., the variation of C-reactive protein, interleukin-6, interleukin-10, cortisol, and lymphocyte subsets count of CD3, CD4, and CD8). All indicators will be tested the day before surgery as well as on the first, third, and fifth day after surgery; (2) the positive rate of malignant cells in the ascitic fluid specimen that will be used to assess the quality of the surgical quality; (3) the positive rate of bacterial culture in the ascitic fluid specimen that will be used to assess the quality of the surgical quality; and (4) general conditions such as postoperative pain score on the first day following surgery using the visual analog score, which will be tested the day after surgery, time to first flatus, time to leave bed postoperatively, time to first liquid diet postoperatively, postoperative hospital stay, operation time, estimated blood loss, and the number of retrieved lymph nodes. The timeline of the measurement of these indicators is shown in Fig. 2.

### Data collection and management

The corresponding investigator in each hospital will gather the medical information and guarantee its accuracy. Moreover, the medical information will be stored in the medical record system. Private information like participants’ names will be erased and replaced by an identification number. Each investigator logs into this system using a private account, and the overall data is only accessible by the principal investigator. An individual blinded to the allocation and surgical processes will conduct the final statistical analysis, and no interim analysis will be performed. As both groups are low-risk intervention, no auditing trial is needed. The sponsor, Taiyuan Li, initiated this trial and has the ultimate authority over this trial. The results of this trial will be shown in the publication.

### Statistical analysis

Non-inferiority analysis of primary outcomes will be performed in the per-protocol population using the chi-squared test with a one-sided significance level of 0.025, in which the power to detect the surgical complication rate difference is 0.8. The per-protocol population refers to cases that completed the corresponding treatment in each group. All analyses will be performed using SPSS 22.0 (IBM, USA). Both primary and secondary outcomes will be analyzed in accordance with the per-protocol principle. Subgroup analyses will be performed on these variables: gender (between male and female), approach (between vagina and rectum), and cT stage. Unadjusted *p* values will be reported but the number of declared subgroups analyses will be specified in all publications. Multiplicative interaction analysis will be applied, as appropriate.

## Discussion

This trial will assess whether robotic NOSES is non-inferior to traditional robotic-assisted surgery for patients with colorectal cancer. Accordingly, the present unpublished retrospective cohort study indicates that colorectal cancer patients who underwent robotic NOSES suffered less pain and passed flatus compared to those who underwent traditional robotic-assisted surgery. Thus, this randomized controlled trial is conducted to systematically analyze the safety and feasibility of robotic NOSES.

In this trial, the surgical complication rate is set to be the primary outcome. In order to testify that robotic NOSES is non-inferior to traditional robotic-assisted surgery for patients with colorectal cancer, many indicators required consummation, such as the survival analysis, which may require a randomized controlled trial. In regard to secondary outcomes, the variations of C-reactive protein, interleukin-6 (IL-6), interleukin-10 (IL-10), cortisol, and lymphocyte subset count of CD3, CD4, and CD8 are employed to assess the surgical response stress and immune response.

The current trial has some inherent limitations. No published studies have demonstrated a concrete complication rate of traditional robotic-assisted surgery, which was calculated based on the retrospective medical record system. However, we augmented the sample size to ensure the detection power.

To the best of our knowledge, this trial is the first to compare the clinical outcomes of robotic NOSES with traditional robotic-assisted surgery. We hope that this trial supports the development of treatment plans for patients with colorectal cancer.

### Trial status

The anticipated recruitment date is February 1, 2020. The estimated completion date is December 31, 2022. Protocol version 1.0, 2019.6.12. If protocol amendments are anticipated, we will update this trial on ClinicalTrials.gov, and the revised protocol will be sent to each investigator.

## Data Availability

The datasets analyzed during the current study are available from the corresponding author on reasonable request.

## Abbreviations

RCT: Randomized controlled trial
NOSES: Natural orifice extraction surgery
ECOG: Eastern Cooperative Oncology Group
